# Neck Circumference as a Predictor of Adiposity among Healthy and Obese Children

**DOI:** 10.3889/oamjms.2015.122

**Published:** 2015-11-25

**Authors:** Nayera E. Hassan, Abeer Atef, Sahar A. El-Masry, Amany Ibrahim, Mones M. Abu Shady, Muhammad Al-Tohamy, Iman H. Kamel, Galal Ismail Ahmed Elashry

**Affiliations:** 1 *Biological Anthropology Department, National Research Centre, Dokki, Cairo, Egypt (Affiliation ID 60014618)*; 2 *Department of Pediatrics, Diabetes Endocrine and Metabolism Pediatric Unit (DEMPU), New Children’s Hospital, Faculty of Medicine, Cairo University, Cairo, Egypt*; 3 *Child Health Department, National Research Centre, Dokki, Cairo, Egypt (Affiliation ID: 60014618)*

**Keywords:** neck circumference, body mass index, children, fat distribution, blood pressure

## Abstract

**BACKGROUND:**

Obesity, particularly in the upper part of body, is a major health problem. Because body mass index (BMI) does not adequately describe regional adiposity, other indices of body fatness are being explored.

**OBJECTIVES:**

To determine if neck circumference is a valid measure of adiposity (fat distribution) among group of Egyptian children.

**SUBJECTS AND METHODS:**

This is a cross sectional study, included 50 obese subjects, aged 7 - 12 years recruited from Endocrine, obesity and Metabolism Pediatric Unit at Children Hospital, Cairo University and 50 healthy children, age and sex matched. All children were subjected to blood pressure assessment (systolic SBP and diastolic DBP), and anthropometric assessment (body weight, height, neck circumference (NC), waist (WC) and hip (HC) circumferences, and skin fold thicknesses at three sites: biceps, triceps and sub scapular. BMI [weight (kg)/height (m2)] was calculated.

**RESULTS:**

In healthy females, significant associations were detected between NC and SBP, DBP and all anthropometric measurements. However, in healthy males NC was not significantly associated with BMI, SBP and DBP. In the obese group; both sexes; insignificant association was found between NC and SBP, DBP, BMI and skinfold thickness.

**CONCLUSION:**

NC is related to fat distribution among normal healthy female children. However, this relation disappears with increasing adiposity. The results do not support the use of NC as a useful screening tool for childhood obesity.

## Introduction

Childhood overweight and obesity is rapidly increasing and remains a worldwide public health concern [1, 2]. Obesity is associated with several risk factors for later cardiovascular and metabolic disturbances. Chronic and insidious nature of these disorders requires close monitoring in childhood to prevent long-term effects. Related to metabolic abnormalities, the determination of inappropriate body fat distribution (upper body>lower body) is significant for metabolic disorders such as glucose intolerance, hyperinsulinemia, diabetes, hypertriglyceridemia, hypertension, and uric calculus disease [3-5].

The most widely used tool for defining overweight and obesity in both adults and children is BMI, which is defined as an individual’s weight in kilograms divided by the square of their height in meters (BMI= kg/m^2^) [6]. Despite the ease of use and popularity of BMI as an anthropometric tool, it is becoming increasingly clear that it is not a good proxy for regional adiposity. Waist circumference and mid-upper arm circumference were defined as a useful indexes to reveal central obesity and were found to be simple screening measures that could be used to identify overweight and obesity [7, 8].

Recent studies in adult, have suggested that measurement of neck circumference, a marker for upper-body subcutaneous fat, might have a complementary clinical value to other body measurements and increased neck circumference surpasses waist circumference as a marker of both visceral obesity and insulin resistance [9, 10]. High neck circumference is associated with a parallel increase in the prevalence of hypertension. Measurement of neck circumference is especially useful in subjects not considered obese by waist circumference measurement [11]. Very few investigators have attempted to use neck circumference to screen for high BMI in children. Therefore, the objectives of this study were to find the correlation between neck circumference and BMI in children, to examine if neck circumference is a valid measure of fat distribution in a group of Egyptian children.

## Subjects and Methods

The study is a cross sectional one conducted on 50 obese subjects (27 male, 23 female), BMI ≥95^th^ percentile for age and sex based on the Egyptian Growth Reference Charts [12], aged 7 to 12 years recruited from Endocrine, obesity and Metabolism Pediatric Unit at Children Hospital, Cairo University. Fifty healthy children (25 male, 25 female), BMI 15^th^ to ‹85^th^ percentile, age and sex matched, were also, included during the period from April 2013 to January 2014. All of them belong to the same social class (low-middle). Ethical approval from ethical committee of both NRC and Cairo University was taken. Written informed consent from one of parents was taken after an explanation of the study before the start.

All children were subjected to history taking, complete clinical examination including blood pressure assessment, and anthropometric assessment (body weight, height, neck circumference (NC), waist (WC) and hip (HC) circumferences, and skin fold thicknesses at three sites: biceps, triceps (peripheral obesity) and sub scapular (central obesity). Children with history of chronic illness, identified syndromes or chromosomal defects or endocrinal disorders causing obesity, chronic use of glucocorticoids, the use of drugs that may affect the blood pressure were excluded from the study.

Blood pressure was measured after the subjects had rested at least 10 min. Three resting BP measurements were obtained from the left upper arm using standard mercury sphygmomanometer and appropriate size cuff. The first measurement was discarded and the average of the other two measurements was recorded as the study visit BP. Systolic blood pressure was recorded at the appearance of sounds, and the diastolic blood pressure was recorded at the disappearance of sounds.

Anthropometric measurements were attempted following the recommendations of International Biological Program [13]. All anthropometric measurements were taken by the same individual who was duly trained for the task. Anthropometric measurements were performed in the morning, before breakfast, with the subject wearing light clothing, without footwear. Body weight was measured using the SECA scale approximated to the nearest 0.5 Kg. Height was measured using Holtain Stadiometer to the nearest 0.1 cm. NC was measured in the midway of the neck, between mid-cervical spine and mid-anterior neck, to within 1 mm, with a flexible non-stretchable plastic tape and approximated to the nearest 0.1 cm, calibrated weekly [14]. The WC was measured at the midpoint between the lowest rib and the iliac crest (the highest point of the ileum) at the end of normal expiration [15], while HC is measured at the maximum circumference over the buttocks. Then, BMI (weight (kg)/height (m) squared) was calculated. The skin fold thicknesses were measured using Holtain skin fold caliper, and approximated to the nearest 0.1 mm.

### Statistical Methodology

Statistical analysis was performed using Statistical Package for Social Sciences (SPSS®) for Windows® version 16.0. Measured data was described as mean and standard deviation (for parametric variables), Difference between two groups was measured using unpaired student’s t-test. Association between variables was assessed using Pearson`s correlation coefficient. *P-value <0.05 was considered significant [16].

## Results

Table 1 shows that the control females had higher values than control males in almost all studied anthropometric parameters, with significant different-ces in hip circumference, biceps skin fold (p < 0.05), triceps skin fold and sub scapular skin fold thickness (p < 0.01). On the other hand, obese females had significantly higher values than obese males in neck circumference, hip circumference, biceps skin fold, triceps skin fold thickness and SBP (p < 0.05), while obese males had significantly higher values in BMI SDS (p < 0.05) (Table 2).

**Table 1:** Sex differences of the healthy group according to sex regarding age and anthropometric parameters

Parameters	Male n = 25		Female n = 25		t	P

	Mean	± SD	Mean	± SD
Age (year)	8.96	1.79	9.68	1.62	-1.49	.143
Clinical						
SBP (mmHg)	100.44	5.85	102.6	5.97	-1.29	.202
DBP (mmHg)	62.20	5.22	64.84	6.11	-1.64	.107
Anthropometric						
Wt. SDS	0.13	0.67	.65	0.54	.36	.721
Ht. SDS	-0.66	0.63	-0.73	0.58	.37	.715
BMI (kg/m²)	18.28	1.97	18.56	1.96	-.49	.622
BMI SDS	0.84	0.77	0.71	0.72	.62	.537
Neck circumference (cm)	28.98	1.66	29.38	1.82	-.81	.422
Waist circumference (cm)	56.84	4.56	59.52	5.76	-1.82	.075
Hip circumference (cm)	67.24	7.53	72.08	7.22	-2.32	.025*
Biceps skin fold (mm)	7.39	2.72	9.28	3.41	-2.16	.036*
Triceps skin fold (mm)	12.61	3.65	15.71	3.95	-2.89	.006**
Sub scapular skin fold (mm)	10.71	3.37	14.11	3.99	-3.25	.002**

Wt. SDS (weight standard deviation score), Ht. SDS (height standard deviation score), BMI SDS (body mass index standard deviation score). SBP systolic blood pressure, DBP diastolic blood pressure, P-value <0.05 was considered significant.

**Table 2:** Sex differences of the obese group regarding age and anthropometric parameters

Parameters	Male n = 27		Female n = 23		t	p

	Mean	± SD	Mean	± SD
Age (year)	9.41	1.95	10.52	1.50	-2.24	.030*
Clinical						
SBP (mmHg)	112.85	7.41	115.65	10.69	-1.06	.297
DBP (mmHg)	73.33	8.99	81.52	12.65	-2.60	.013*
Anthropometric						
Wt. SDS	3.26	1.77	2.73	.97	1.347	.185
Ht. SDS	-.51	1.20	-.47	.84	-.13	.901
BMI (kg/m²)	29.70	2.87	30.14	3.56	-.48	.635
BMI SDS	3.29	.67	2.89	.39	2.64	.012**
Neck circumference (cm)	32.90	1.63	33.87	1.47	-2.18	.033*
Waist circumference (cm)	88.11	7.66	89.32	8.99	-.52	.608
Hip circumference (cm)	91.74	8.95	98.76	10.58	-2.54	.014**
Biceps skin fold (mm)	17.33	4.01	20.17	5.11	-2.20	.033*
Triceps skin fold (mm)	24.52	4.45	27.22	4.91	-2.04	.047*
Sub scapular skin fold (mm)	24.89	5.03	26.70	4.9	-1.28	.207

Wt. SDS (weight standard deviation score), Ht. SDS (height standard deviation score), BMI SDS (body mass index standard deviation score). SBP systolic blood pressure, DBP diastolic blood pressure, P-value <0.05 was considered significant.

Among control group (Table 3), for both sexes; there were significantly positive correlation between neck circumference and weight, height, waist, hip circumferences, biceps, triceps and sub scapular skin fold thickness. In addition; control females had significant positive correlation between neck circumference and BMI, systolic and diastolic blood pressure (p < 0.01).

**Table 3:** Correlation between neck circumference and other parameters among healthy subjects

Parameters	Healthy males		Healthy Females	

	r	p-value	r	p-value
Clinical				
SBP (mmHg)	.261	.208	.700**	.000
DBP (mmHg)	.088	.677	.568**	.003
Anthropometric				
Weight (kg)	.655**	.000	.828**	.000
Height (cm)	.749**	.000	.799**	.000
BMI (kg/m²)	.184	.379	.685**	.000
Waist circumference (cm)	.750**	.000	.483*	.015
Hip circumference (cm)	.824**	.000	.673**	.000
Biceps skin fold (mm)	.493*	.012	.531**	.006
Triceps skin fold (mm)	.598**	.002	.453*	.023
Sub scapular skin fold (mm)	.623**	.001	.535**	.006

* = significant;

** = highly significant.

However; among obese group (Table 4); the correlations between neck circumference and the skin fold thickness at the three sites disappear for both sexes, and those between neck circumference and either BMI or diastolic blood pressure among obese females.

**Table 4:** Correlation between neck circumference and other parameters among obese subjects

Parameters	Obese Males (N = 27)		Obese Females (N = 23)	

	r	p-value	r	p-value
Clinical				
SBP (mmHg)	-.093-	.644	.307	.155
DBP (mmHg)	.219	.272	-.103-	.641
Anthropometric				
Weight (kg)	.551**	.003	.548**	.007
Height (cm)	.517**	.006	.671**	.000
BMI (kg/m²)	.316	.109	.231	.288
Waist circumference (cm)	.490**	.010	.593**	.003
Hip circumference (cm)	.466*	.014	.560**	.005
Biceps skin fold (mm)	.165	.412	-.298-	.167
Triceps skin fold (mm)	.155	.442	.357	.094
Sub scapular skin fold (mm)	.189	.346	.190	.386

* = significant;

** = highly significant.

Among obese males and females, there were significantly positive correlations between neck circumference and weight, height, waist and hip circumferences only.

## Discussion

The prevalence of obesity in children has increased worldwide [1] and is associated with risk factors for cardiovascular and metabolic disorders, which, due to their chronic and insidious nature, require careful monitoring in childhood, aimed at early detection and the establishment of interventions to prevent complications in adulthood [17, 18].

In adults, it is well-determined that a more central fat distribution is associated with an increased risk of metabolic diseases. Recently, it has also been shown in children that a greater deposition of central fat is correlated with hypercholesterolemia and hypertension. Thus, it should be important to determine upper body fat rather than total body fat. Direct measurement of body fat content and distribution, e.g., dual X-ray absorpsiometry, bioimpedance, hydrodensitometry, is used as accurate measure of obesity, but these methods are neither practical nor inexpensive [19,20].

NC may be used to assess upper fat distribution, especially for screening purposes, as an easy and practical anthropometric index. It is more practical and even easier to perform than the measurement of WC. Additionally, NC shows very good inter and good intra-rater reliability, which does not require multiple measurements for precision and reliability compared with WC [21].

In the present study, no significant difference was detected between males and females as regard to NC in the healthy group, while in the obese group, a significant higher value of mean NC was found in females than in males. In healthy females, significant associations were detected between NC and SBP, DBP and all anthropometric measurements including BMI and waist circumference. However, in healthy males NC was not significantly associated with BMI, SBP and DBP. Nevertheless, in both sex in the obese group no significant association was found between NC and BMI and significant association was detected between NC, waist and hip circumferences.

Regarding the NC, despite the scarcity of studies in the literature that adopted this measurement, the results those that used it as a parameter to assess central adiposity in children indicate that such measurement may be a useful screening tool to identify overweight or obesity. It may also be useful to diagnose children at risk for high adiposity, an important predictor of cardiovascular health problems [22-27]. Ferretti et al., concluded that NC was a great screening measure for identifying overweight in clinical practice, as well as having all the advantages of the ease of measurement, showed an association with other risk factors for chronic diseases [28]. In all the previously mentioned study, the CDC growth charts for BMI may not be accurate enough to serve as a reference method for developing a precise set of NC cut-offs. While BMI has been considered a useful screening tool for epidemiological studies with large sample sizes, it tends to yield biased estimates of total fat distributions at an individual level (dual-energy X-ray absorptiometry for body composition measurement is the gold standard) [29], thereby limiting the practice of BMI as a “gold standard” measure in identifying overweight/obese children. This may have impaired the accuracy of the NC cut-offs developed in the previously mentioned studies.

Although NC measurement is inexpensive, and easier to obtain than other markers of adiposity (WC and BMI), and has good inter-rater reliability, the results of the present study showed that it performed unwell as an index of high BMI in the children of both sexes in obese group and in male healthy; therefore, NC could not be a useful screening instrument for identifying overweight or obese children. In agreement with our results, Kim et al., concluded that NC was inferior to BMI. Pediatricians and/or pediatric researchers should be cautious or wary about incorporating NC measurements in their pediatric care and/or research [30].

A study of Kuciene et al. evaluated the associations between high NC (neck circumference) alone and in combinations with BMI (body mass index), WC (waist circumference), and high BP among Lithuanian children and adolescents aged 12 to 15 year. They detected an association between high NC alone particularly in combinations with overweight/obesity and abdominal overweight/obesity with an increased risk of high BP which is in concordance with the result of the present study as regard to female control group [31].

In conclusion: NC is related to fat distribution among normal healthy female children. However, this relation disappears with increasing adiposity. The results of this study appear not to strongly support the use of NC measurement as a useful screening tool for classifying childhood overweight/obesity. While NC measurement holds great practicality, its unsatisfactory accuracy in overweight/obesity classification may preclude the widespread use at clinical settings. In order for NC measurement to be widely adopted in clinical practice, therefore, additional studies are needed to develop and/or to evaluate a set of NC cut-offs relative to a gold-standard reference (i.e., Bod Pod, dual-energy X-ray absorptiometry) for body composition measurement with average populations of children.

Our study has several limitations that need to be addressed in future research. The sample size was small consisting of young healthy and obese children and, therefore, the results cannot be generalised over the whole population. The current study is a cross sectional, examined only a sample of 7–12 year-old children. Therefore, our findings need to be confirmed and extended in further larger or collaborative studies among children of wider age group.
